# Supplementary material for: Optimizing management of low back pain through the pain and disability drivers management model: A feasibility trial
Source: PLoS One. 2021 Jan 20;16(1):e0245689. doi: 10.1371/journal.pone.0245689 (PMC7817044; doi:10.1371/journal.pone.0245689)
Supplement: S3 Table — (DOCX) [file pone.0245689.s003.docx]

We used the following method to establish the patient’s profile (categorization) according to the PDDM framework. The table below describes, for each domain, which clinical characteristics needed to be present/absent in order to categorize a patient within one of the four (4) possible categories:

(A) Presence of at least one element of the Category A,

(B) Presence of at least one element of the Category B,

(A+B) Presence of at least one element of the Categories A and B

(0) Absence of elements in either A or B categories.

| **Domain 1 : Nociceptive pain drivers** |
| --- |
| 0: The patient reports no symptoms of pain.  **A:** ***Responder to classification system***  Pain is mechanical and upon physical examination, the patient CAN be classified into one of the 3 subgroups of the Treatment-Based Classification system: i) symptom modulation, ii) movement control, iii) functional optimization.  **B: *Non-responder to classification system***  Pain is mechanical and upon physical examination, the patient CANNOT be classified into a subgroup of the Treatment-based classification system (non-responder).  **OR**  The patient seems to suffer from an active inflammatory process (e.g., ankylosing spondylitis) AND/OR the symptoms are not in relation with any mechanical pattern.  **OR**  The patient had a recent (less than three months) lumbar surgery or trauma (e.g., fracture). |
| **Domain 2: Nervous system dysfunction drivers** |
| 0: The patient reports no symptoms of pain or the therapist observes no signs of nervous system dysfunctions.  **A: *Peripheral sources of nervous system dysfunctions***  The patient shows clinical evidence of neuropathic pain such (i.e., symptoms of burning pain, electrical shock) which irradiate within a radicular pain pattern AND/OR paresthesia/tingling in a specific dermatome.  **OR**  The patient presents with radiculopathy or myelopathy by the presence of at least one positive sign on neurological assessment:   - Myotomes (loss of strength) - Dermatomes (loss of sensitivity) - Loss of motor reflex and/or neurogenic claudication   **B: *Nervous system hypersensitivity***  The physical examination revealed evidence of hypersensitivity by the presence of at least one of the following:   - Allodynia - Evidence of disproportionate pain intensity - Hyperalgesia - Widespread pain - Sympathetic nervous system dysfunctions - A score > 40 on the Central Sensitization Inventory   **OR**  The patient presents evidence of increased neural mechanosensitivity (i.e., positive SLUMP, PKB or SLR).  **OR**  Painful symptoms provoke significant sleep disturbances with negative impact on patient’s well-being. |
| **Domain 3: Comorbidity drivers** |
| 0: No relevant comorbidity.    **A: *Physical Comorbidities***  The patient reports ongoing painful comorbidity factors related to the musculoskeletal system (i.e., osteoarthritis, rheumatoid arthritis, tendinopathy, active soft tissue lesions).  **OR**  The patient reports a diagnosis/pathology related to chronic pain such as:   - *Fibromyalgia* - *Restless legs syndrome* - *Migraine* - *Complex regional pain syndrome* - *Irritable bowel syndrome*   **B*: Mental-health comorbidities***  The patient currently has sleep disorders unrelated to the current painful condition such as**:**   - Insomnia - Sleep apnea   **OR**  The patient reports a diagnosis (active) of a mental health- disorder such as:   - Depressive disorder - Anxiety disorder - Personality disorder - Substance abuse - PTSD - Other DSM-5 diagnoses |
| **Domain 4: Cognitive- emotional factors** |
| O: The patient has a score <4 on the Start Back Tool AND has no relevant maladaptive behaviors.  **A: *Cognitive-emotional factors***  The patient has a score > 3 on the Start Back Tool.  **AND**  The assessment (subjective and physical examinations) highlights at least 2 "maladaptive" elements from the cognitive-affective domain among   - Pain-related anxiety - Pain-related fear OR kinesiophobia - Maladaptive beliefs about pain - Passive or harmful coping - Low level of understanding of one's health condition - Low perceived treatment efficacy - Low self-efficacy - Perception of injustice related to his situation - Negative anticipation of the evolution of his condition - High level of catastrophization - Strong perception of "being sick"   **B: *Maladaptive pain-behaviors***  The patient exhibits maladaptive behaviors related to pain such as**:**   - Avoidance of certain tasks/movement - Facial expressions (e.g., grimacing, wincing during movement) - A guarded posture - Moving as a block - Verbal/paraverbal pain expressions (e.g., grunts, sighs) |
| **Domain 5 : Contextual drivers** |
| 0: No occupational or social factors identified.    **A:** **Occupational context**  Score on the Örebro Musculoskeletal Pain Questionnaire >49.  **OR**  The patient is on sick leave (total or partial) (or unable to participate in one of his occupations if he is retired or unemployed) AND foresees obstacles for his return to work/occupation such as:   - *Rigid internal return-to-work regulations* - *Perception of heavy work* - *High job stress* - *Low job satisfaction*     **B**: S**ocial context**  The patient CANNOT rely on the presence of adequate social support such as:   - Support from family and friend - Professional support from employer and colleagues - Caregivers facilitating his rehabilitation process with a positive and empathetic attitude towards his condition   **OR**  The patient is confronted with at least one (1) element related to the social context among the following:   - Negative experience during a previous episode of care - Restricted or non-existent access to the required care - Communication barriers (e.g., language, culture, difficulty telling his medical history) |
