# Supplementary figures and images for: Optimizing management of low back pain through the pain and disability drivers management model: A feasibility trial
Source: PLoS One. 2021 Jan 20;16(1):e0245689. doi: 10.1371/journal.pone.0245689 (PMC7817044; doi:10.1371/journal.pone.0245689)

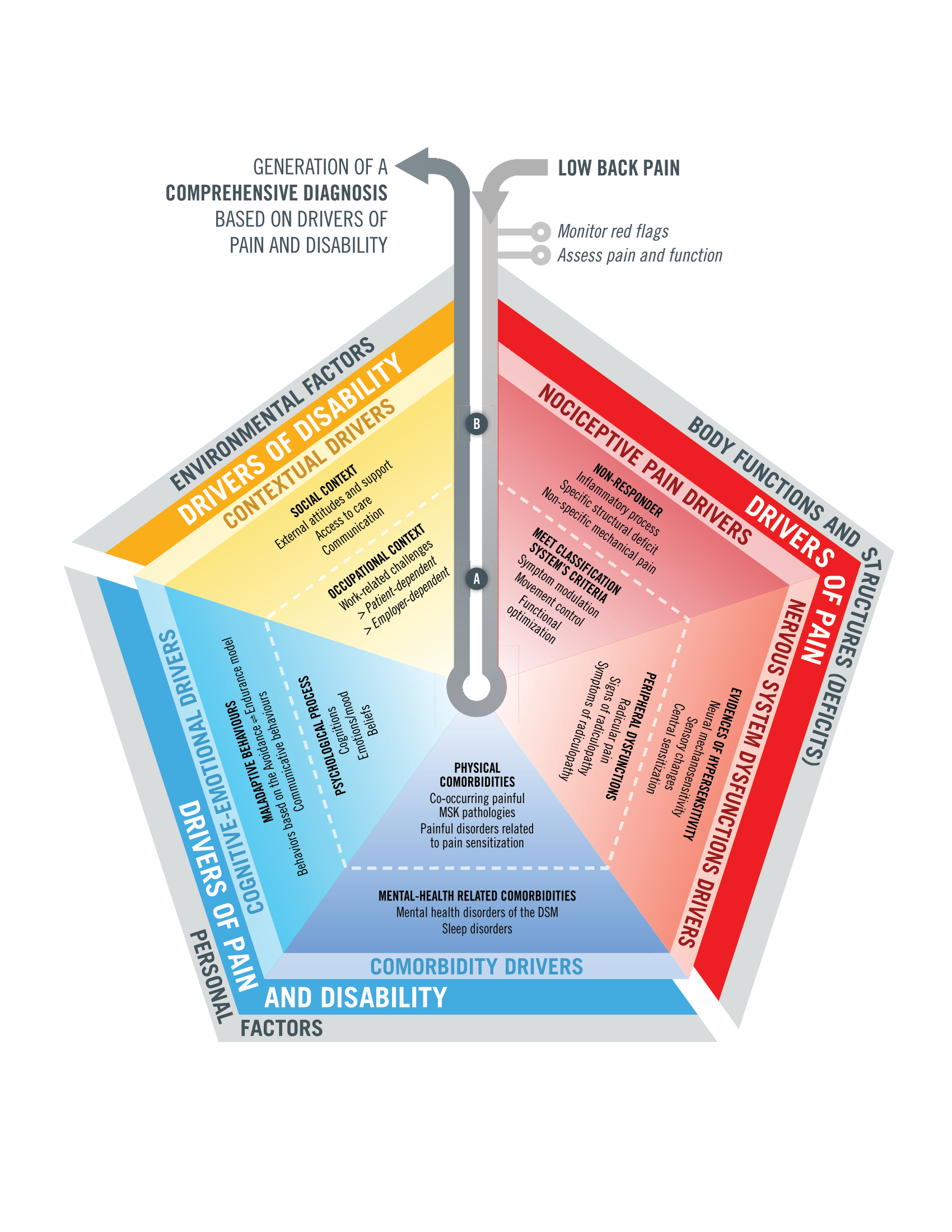

Supplement: S1 Fig — (TIF) [file pone.0245689.s004.tif]

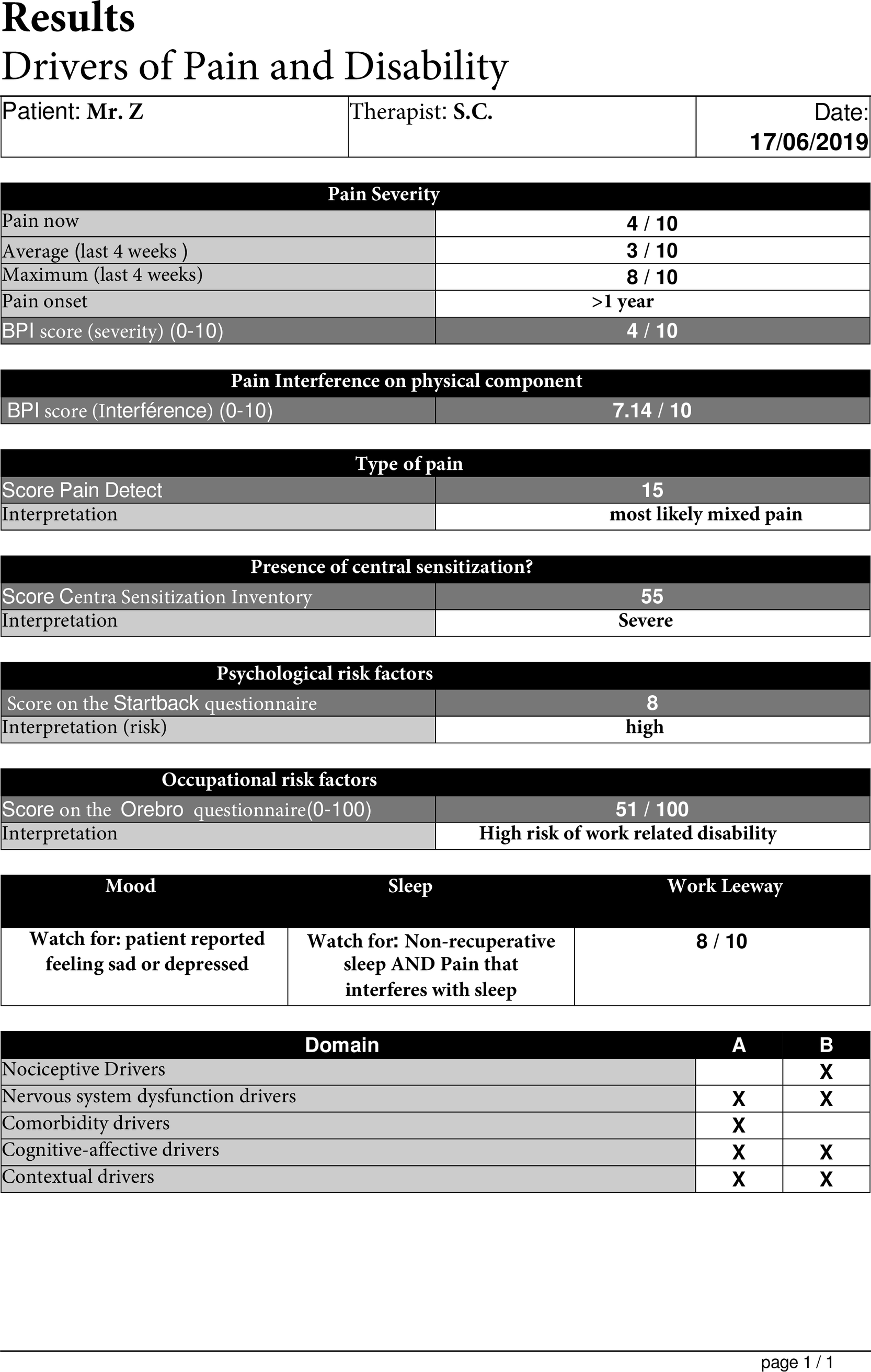

Supplement: S2 Fig — (TIF) [file pone.0245689.s005.tif]

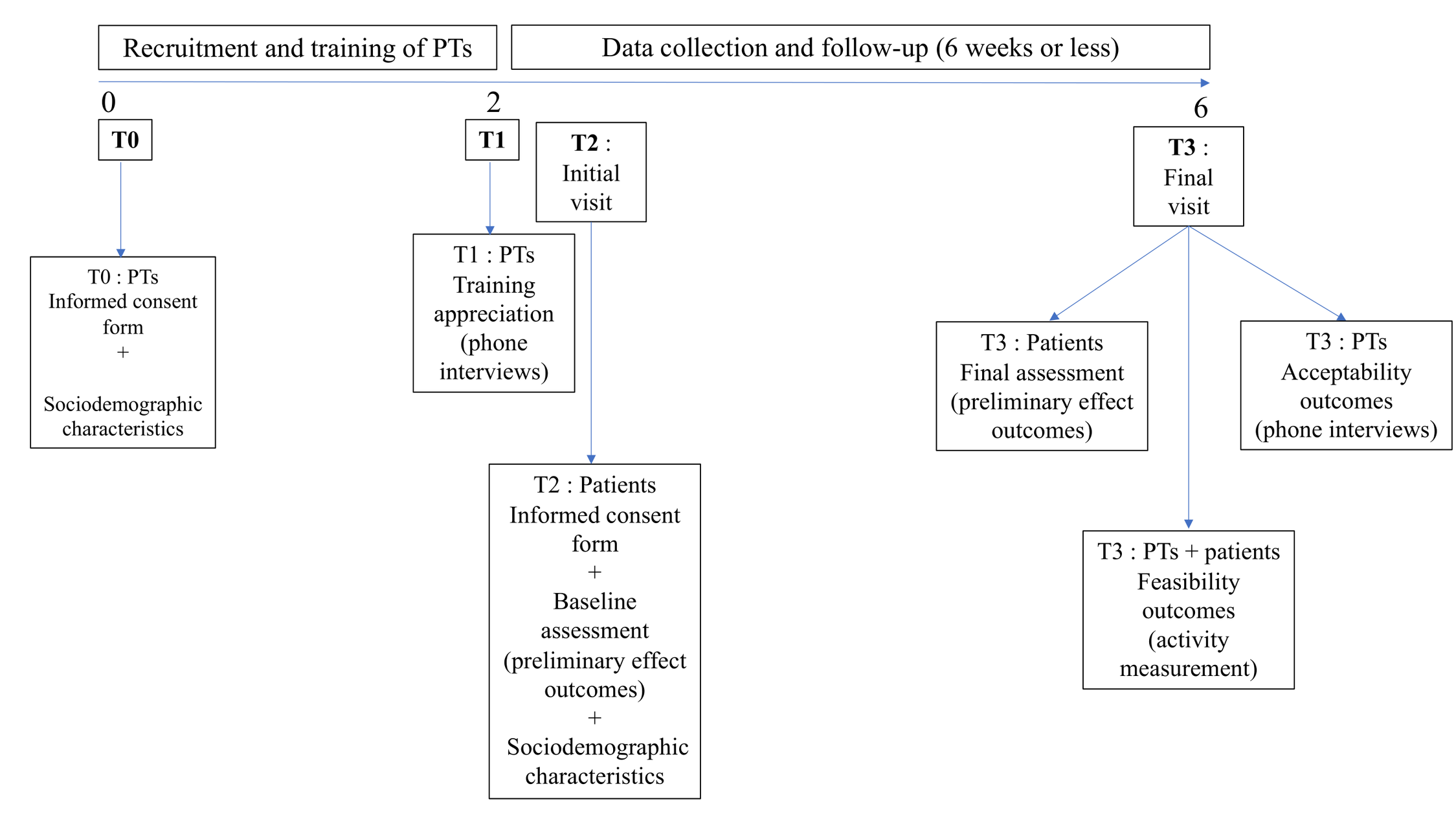

Supplement: S3 Fig — (TIF) [file pone.0245689.s006.tif]
