# Supplementary material for: Optimizing management of low back pain through the pain and disability drivers management model: A feasibility trial
Source: PLoS One. 2021 Jan 20;16(1):e0245689. doi: 10.1371/journal.pone.0245689 (PMC7817044; doi:10.1371/journal.pone.0245689)
Supplement: S1 Appendix — (PDF) [file pone.0245689.s007.pdf]

Training’s appreciation (T1):

1. Does the functioning of the model make sense to you?
    a. How does it make sense? In what way does it influence your clinical practice?
2. What did you think of the course of the workshop?
    b. Regarding the order of presentation? The duration of the workshop? The content of the workshop (amount, quality)?
3. In your opinion, what are the strengths of the training?
    c. What helped you the most during the workshop?
4. In your opinion, what would be the specific aspects of the training to be improved?
    d. Course of the training, physical setting, content, etc.
5. Do you have any suggestions to improve the general course of the training?
6. What did you think of the resources presented to you during the training?
    e. Are they relevant? Do you plan to use them? Do you think they will help you apply the model?

Experience with the electronic data collection tool LimeSurvey® and online support resources (T3):

1. Please tell me about your experience with the LimeSurvey® platform?
    a. Comprehensibility, applicability, interpretation, ease of use, time needed to complete the assessment.
2. Have you used the online support resources offered (i.e., website, questionnaires)?
    a. If yes, were they relevant? Did they help you apply the model into your practice?
    b. If we were to develop other similar resources, what would your needs be in terms of online resources?
