# Supplementary material for: Optimizing management of low back pain through the pain and disability drivers management model: A feasibility trial
Source: PLoS One. 2021 Jan 20;16(1):e0245689. doi: 10.1371/journal.pone.0245689 (PMC7817044; doi:10.1371/journal.pone.0245689)
Supplement: S2 Appendix — (PDF) [file pone.0245689.s008.pdf]

Perception of the PDDM model’s contribution (T3):

1. Has the PDDM model contributed to your patient's assessment?
    a. If yes, can you explain to me how the model has contributed to your patient’s assessment?
2. Following your patient’s assessment, has the PDDM model contributed to establish a treatment plan with your patient?
3. Has the PDDM model contributed to your patient's assessment?
    b. If yes, can you explain to me how the model has contributed to your patient’s assessment?
4. Following your patient’s assessment, has the PDDM model contributed to establish a treatment plan with your patient?
