# Supplementary material for: Optimizing management of low back pain through the pain and disability drivers management model: A feasibility trial
Source: PLoS One. 2021 Jan 20;16(1):e0245689. doi: 10.1371/journal.pone.0245689 (PMC7817044; doi:10.1371/journal.pone.0245689)
Supplement: S1 Protocol — (DOCX) [file pone.0245689.s009.docx]

**Optimizing management of low back pain through the Pain and Disability Drivers Management Model: study protocol for a feasibility trial**

Christian Longtin, PT^1^, Simon Decary, PhD^3^, Marc O. Martel, PhD^2^, Sylvie Lafrenaye, PhD^4^, Carlesso, Lisa, PhD^5^, Yannick Tousignant-Laflamme, PhD^1,6^

*^1^School of Rehabilitation, University of Shebrooke, Sherbrooke, QC, Canada;*

*^2^Faculty of Medecine, Laval University, Quebec, QC, Canada;*

*^3^Faculty of Medecine, McGill University,, Montreal, QC, Canada;*

*^4^Faculty of Medecine, University of Sherbrooke, Quebec, QC, Canada;*

*^5^School of Rehabilitation, University of Montreal, Montreal, QC, Canada;*

*^6^Research Centre of the CHUS, CIUSSS de l’Estrie-CHUS, Sherbrooke, QC, Canada;*

Corresponding author’s name, address and email address:

Yannick Tousignant-Laflamme

[Yannick.Tousignant-Laflamme@usherbrooke.ca](mailto:Yannick.Tousignant-Laflamme@usherbrooke.ca); 819-821-8000, ext 72912

School of Rehabilitation, Faculty of Medicine and Health Sciences, 
Université de Sherbrooke, 3001 12e avenue Nord, Sherbrooke, QC J1H 5N4

**Abstract**

**Background:** Non-specific mechanical low back pain is highly prevalent, recurrent and is a leading cause of disability worldwide. Despite increased efforts in improving care, the self-reported levels of disability in individuals with LBP have not improved in the last decade. In order to more effectively manage LBP through non-pharmacological approaches, evidence endorses the use of classification systems to support diagnosis and guide treatments. However, this approach to care is not without limitations and a more comprehensive and broader perspective is needed. Hence, we recently proposed and validated the Low Back Pain and Disability Drivers Management (PDDM) model, which aims to identify the domains influencing pain and disability to create an ICF-based profile or phenotype to guide clinical decisions. The objective of this study is to assess the feasibility of conducting a trial and to gather preliminary effect outcomes of the intervention in clinical setting.

**Methods:** The design is a prospective single arm experimental design. Physiotherapists (PTs) working with a population suffering from musculoskeletal disorders and patients presenting with non-specific mechanical LBP from two different clinical settings will be recruited. The intervention consists of a one-day training workshop for PTs on using the PDDM to guide management decisions. PTs will then perform a 6-weeks follow-up with their participating patients. This feasibility study will assess recruitment and retention rates as well as PTs’ acceptability and patients’ satisfaction outcomes related to the intervention. Preliminary effect outcomes will also be gathered.

**Discussion:** This study will inform the feasibility and acceptability of the intervention and gather preliminary outcomes to conduct a future randomised controlled trial to measure the effectiveness of the intervention in managing non-specific mechanical LBP.

**Keywords: Low back pain, Pain management, Rehabilitation, Feasibility outcomes**

**Background**

Non-specific mechanical low back pain (LBP) is defined as “a symptom for which we are currently unable to reliably identify the pathology” [1]. This painful musculoskeletal problem is highly prevalent (up to 84% lifetime prevalence), recurrent [2] and is a leading cause of disability in both high- and low-income countries [3]. Despite increased awareness for health systems and decision makers in improving care, the self-reported levels of disability in individuals with LBP have not improved in the last decade [4]. Managing disability is crucial since it is a strong predictor of chronicity [5] and has important negative social impacts and costs.

Studies have shown that factors involved in the development of persistent LBP-related disability include psychological, biological, social, and environmental influences [5, 6]. Since disability management in rehabilitation focuses on functioning, the International Classification of Functioning, Disability and Health (ICF) framework is preferred by medical and rehabilitation professionals, as it represents a biopsychosocial framework that provides an overall view of the different aspects of health (biological, individual, and social) [7, 8]. It could serve as the foundational underpinning of a comprehensive framework to better guide the rehabilitation management of LBP.

In order to more effectively manage LBP through rehabilitation approaches, evidence endorses that rehabilitation professionals use classification systems (CS) to help formulate their diagnosis and guide treatments [9]. While CS have improved our understanding of LBP profiles and risk stratification, they have shown limitations. We must recognize that the classification systems currently available CS for the management of LBP 1) mostly focus on addressing deficits related to biological aspects explaining pain and disability and 2) poorly integrates contextual factors driving the experience of pain, such as the person’s environment (social, physical) and inherent personal factors [10]. These aspects can lead to 1) an incomplete clinical profile, 2) inadequate or inappropriate specific relevant interventions (i.e., providing thrust manipulations alone to a patient presenting high levels of cognitive-affective factors) which ultimately lead to modest clinical outcomes [10, 11]. Currently, the use of CS alone can create diagnostic uncertainty which can be define as a perception of lack of knowledge that can change overtime and impedes the clinician’s ability to initiate definitive treatment for a stated problem [12], lead to generic versus personalized care, and put unnecessary stress on already limited resources [10, 11]. Hence, a more comprehensive and broader perspective is needed.

With that in mind, we recently proposed and validated a non-pharmacological management model (The Low Back Pain and Disability Drivers Management (PDDM) model) that encompasses the multidimensional elements included within the ICF framework [13]. Detailed description of the model is presented elsewhere [13]. Our model is distinct from previous research in this area, as it does not propose a new CS. Rather, the PDDM build upon decades of LBP research to provide the first theory-informed guide to improve clinical decision-making processes. It aims to identify the domains influencing pain and disability to create an ICF-based profile or phenotype to guide clinical decisions. Our model is composed of five domains upon which the clinician can base his assessment and orientate treatment allocation and includes: 1) nociceptive pain drivers (i.e., somatic, inflammatory or mixed pain), 2) nervous system dysfunction (NSD) drivers (i.e., sensitization of the peripheral and/or central nervous system), 3) comorbidity drivers (i.e., physical and/or mental health comorbidities), 4) cognitive-emotional drivers (i.e., maladaptive cognitions and/or behaviors) and 5) contextual drivers (i.e., occupational-related and social environmental contextual drivers) [13]. Each domain contains two categories, the first (A) implicates more common and/or modifiable factors, whereas the second (B) involves more complex elements. Patient management should be individualized to the combined contribution of each domain driving the experience of pain and disability. Thus, this profiling will inform and lead the clinician’s treatment approach.

Ultimately, we hypothesize that such a theoretical framework will help rehabilitation clinicians provide more targeted care, optimize treatment outcomes as well as resource utilization in the management of LBP. As our newly developed model has shown sufficient face validity to potentially guide the non-pharmacological management of LBP [14], the next step is to assess its feasibility with clinicians and a sample of patients presenting with non-specific LBP. Although we’ve previously laid the conceptual components of the model and identified the optimal tools to assess the elements included within each domain of the model [14], we have yet to gather data in regard to its feasibility (acceptance, barriers and facilitators to the clinical use of the model) and preliminary effects on clinical outcomes. Hence, prior to implementation and assessment of its effectiveness through a multisite randomized control trial (RCT), we need to gather pilot data regarding these important aspects.

**Objectives**

The general objective of this project is to assess the feasibility of conducting a complete RCT to evaluate the impact of the PDDM model on improving the management of non-specific LBP. The specific objectives are:

- To evaluate the feasibility of the PDDM to guide assessment and treatment;
- To document the clinicians’ acceptability and patient satisfaction of its use in clinical settings;
- To explore the model’s short-term effect on patient’s clinical outcomes (gather preliminary evidence of change in levels of pain and disability and quality of life).

**Methods**

**Design**

A prospective single arm design will be carried out to test the clinicians’ acceptability and the feasibility of the intervention for people with non-specific LBP as well as to gather preliminary data on its effectiveness through quantitative and qualitative methods. The CONSORT statement extension for feasibility trials [15] and the SPIRIT statement list [16] guided the development of this feasibility study protocol.

**Study setting**

Participants (clinicians and patients) will be recruited from two different orthopedic physiotherapy clinics: the orthopedic physiotherapy outpatient clinic of the Centre Hospitalier Universitaire de Sherbrooke (CHUS) and private physiotherapy clinics from the network of clinics of *PhysioExtra* in the greater region of Montreal, Quebec. These clinical settings have already expressed interests in participating and we’ve already established collaborations with the physiotherapists (PT) from the two sites from past research projects. Also, they have access to a large population of patients suffering from musculoskeletal disorders (MSD), such as non-specific LBP. Moreover these 2 settings representing both the private and public sectors of the healthcare system in Quebec will contribute to the external validity of the study.

**Participants**

To be included, PTs will have to 1) be working with a population suffering from MSD such as LBP and have a valid license to practice physiotherapy in the province of Quebec, 2) agree to participate to the one-day training workshop (intervention), 3) assess and initiate treatment of their patients presenting with non-specific LBP guided by our newly developed model. Patients presenting with non-specific LBP according to the assessment conducted by the PT will be eligible to participate in the study and will have to agree to provide patient related-outcomes obtained following their assessment/treatment in physiotherapy.

**Recruitment**

A convenience sampling method will be used to recruit the clinicians. PTs from the two sites that express interest in participating in the study will be enrolled if they meet the inclusion criteria. An email containing all the relevant and necessary details to participate in the study including the participant’s (PTs) implication and the research team coordinates will be sent to all the clinicians from both sites (see [Appendix A](#_Annex_A:_invitation)). Patients participating in the study will be recruited based on the participating clinicians’ caseload. Each PT will have to recruit participants (new patients) by explaining in detail the purposes, process and implications of participating in the study. A formal consent form with all the aforementioned details will be provided by the PT to the patient. As this is a feasibility trial, no formal sample size calculation was conducted [17]. Instead, we aim to recruit between 10-15 PTs in order to effectively measure the feasibility of implementation of the intervention in a 3-4 months timeframe. We aim to have each PT provide complete data for at least five of their patients with non-specific LBP for an expected total of 50-75 patients.

**Intervention**

All participants (PTs) will undergo training to acquire specific skills tenant to the model. Training will be provided by the lead investigator (YTL) with support from co-investigators (CL, MOM, SD). YTL has developed the structure and content of the intervention which has been pre-tested in a continuing education context with licensed PTs in the province of Quebec, Canada. PTs’ integration of the PDDM model into their practice will also be supported by the development of a website to facilitate the use of resources (e.g. short educational capsule for PTs, treatment algorithms).

The objectives of the workshop are: 1) to acquire extensive knowledge on the functioning of the PDDM model by identifying the different domains of the model, and the specific elements that are deemed “problematic” for a given patient. This will allow the therapist to appropriately establish the clinical profile of the patient based on the relative contribution of each domain and 2) to adopt a structured approach to manage and select appropriate interventions to address problematic areas. The training lasts 8 hours (one-day workshop format) and its content includes a presentation of the model to facilitate the integration and operationalization of the different concepts (part 1) followed by the exploration of different intervention strategies based on the clinical profile of the patient supported by the presentation of two case studies (part 2) (see [Appendix B](#_Annex_B_:)).

Following the training, we estimate that adequate knowledge and skills will have been provided to the participating PTs, enabling them to deliver the intervention to their patients based on the principles of the PDDM model. For each of their patients, the PT will have to provide clinical data based on their use of the PDDM model to guide their assessment and interventions over a 6-weeks period (or less if the patient is discharged before the end of the 6-week period). The data collected relates to the selected outcomes to measure feasibility, acceptability and preliminary effect of the intervention. Concerning the specific use of the PDDM model in order to guide the clinicians’ assessment and treatment approach, an electronic tool using Excel will be developed by the research team. This tool aims to foster the identification by the clinicians of the relative contribution of each domain to the patient health condition by creating a complete personalized profile and to gather baseline patients’ sociodemographic characteristics. This tool will be presented to the PTs during the training seminar.

**Outcomes**

The primary outcomes measures will assess feasibility of implementation, clinician’s acceptability and patient’s satisfaction of the intervention. These outcomes will be measured at T0 (i.e., before the workshop), T1 (i.e., after the workshop), T2 (i.e., at patient’s initial visit/enrollment) and T3 (i.e., after follow-up period) (see Figure 1). Sekhon et al. defined acceptability as a “multi-faceted construct that reflects the extent to which people delivering or receiving a healthcare intervention consider it to be appropriate” [18]. It includes several constructs such as attitude towards the intervention, the intervention’s burden and ethicality for the individual, the intervention coherence, perceived effectiveness and self-efficacy in performing the intervention. Feasibility refers to the assessment of whether or not it will be feasible to conduct an intervention in a particular setting and includes issues such as willingness of individuals to participate in the study, the adherence/compliance of participants to the intervention (ex : response rate to questionnaire, loss to follow up) and whether the intervention can be delivered as intended within the clinical setting [19]. Despite the fact that there is some overlap between these constructs, we decided to use specific data collection procedures for each aspect included in these constructs.

**Feasibility outcomes**

The feasibility outcomes include:

1. Recruitment rate: % of eligible clinicians (T1) and patients who enrolled in the study (T2)
2. Retention rate: % of contacted clinicians and patients accepting to participate and report data (T3);

**Acceptability outcomes**

The clinician’s acceptability outcomes will be assessed via questionnaires and semi-structured phone interviews and include:

1. Clinician’s perception of suitability of the assessment procedures to refine the diagnosis label will be assessed at T3 via a brief phone interview: During your assessment with this patient, was the PDDM useful to:
   1. Establish a complete clinical profile of your patient?
   2. Reduce diagnostic uncertainty?
   3. Identify all the factors driving pain and disability to refine your clinical diagnosis?
2. Clinician’s perception of suitability of the assessment procedures to target adequate treatment will be assessed at T3 via a brief phone interview: After your assessment, was the PDDM useful to improve decisions points related to:
   1. Establish the specific treatment needs of your patient (target care)?
   2. Refer out to other health care providers if indicated (interdisciplinary collaboration)?
   3. Prioritize care (importance and timing)?
3. Clinician’s attitudes and beliefs concerning the management of pain will be assessed before and after the training and at the end of the data collection period (T0, T1 and T3) via a self-administered questionnaire, the *Pain Attitudes and Beliefs Scale for Physiotherapists* (PBS-PT) [20] in order to explore the effect of the training on this variable ;
4. Clinician’s appreciation of clinical training (assessed within a week after the training, T1) and appreciation of the web-based support (assessed at T3) will be measured via phone interviews to collect their opinion on the strengths, limitations and suggestions on how to improve the quality of the various content elements of the training via open-ended questions (see [Appendix C](#_Annex_C_–)).
5. Additional time required to complete assessment and guide treatment (T3);
   1. Compared to a routine assessement, how much extra time do you need to perform an assessment based on the tenants of the PDDM model?

**Patient Satisfaction outcomes**

Patients’ satisfaction towards the clinical encounter outcomes with their therapist will be assessed through a survey distributed upon discharge or after the 6-weeks data collection period of the study (T3). The Visit-Specific Satisfaction instrument (VSQ-9) [21] consists of a 9-item self-report questionnaire, which aims to rate the overall satisfaction of the patient toward his clinical encounter with a healthcare professional. The patient has to rate each item on a scale from “poor” to “excellent”. The score is then transformed linearly to a 0 to 100 scale, with 100 corresponding to “excellent” and 0 to “poor”. Then, a VSQ-9 score for each person is created based on the average response score on all of the 9 items. An adapted version will be used according to the context of the study (see [Appendix D](#_Annex_D)).

**Preliminary effect outcomes**

The secondary outcomes measures will assess the preliminary effects of the intervention. We will first obtain sociodemographic characteristics (i.e. age, sex, educational level, clinical setting, completed continued education in the last 5 years) of the participating PTs at the training seminar using self-administered questionnaires. We are also going to determine the percentage of patients that fall into the different subcategories of the model. This will allow us to explore all possible subgroups of patients based on the PDDM.

The documentation of the preliminary short-term (T2: initial visit; T3: final visit, +6 weeks) clinical effects of the use of the model will be assessed through the analysis of the core outcome measures for the five domains of the PDDM (see Table 1). Dyads (PTs and patients) will have to complete the questionnaires below and enter all the relevant data in the electronic survey (Excel). The number of visits will also be reported during the treatment period. The participating PTs’ patients will have to complete baseline assessments (T0) and final assessment (T1) based on the outcome measures identified related to the five domains of the PDDM model and a sociodemographic and health questionnaire (age, sex, comorbidities, medication) already used in both clinical settings. Following their training, the PTs will have the adequate knowledge to perform the assessment and support the patients in completing the questionnaires.

**Table 1. Outcome measures of the five domains of the PDDM model**

| **Domain** | **Specific items** | **Outcome measures (T2 and T3)** |
| --- | --- | --- |
| Nociceptive pain drivers | Pain and impact of pain on physical function | ***Brief Pain Inventory*** (BPI) is designed to assess pain intensity (at its worst, least and average) and the extent to which pain interferes in the daily life in relation to 7 domains of functioning (general activity, mood, walking ability, normal work, relations, sleep and enjoyment of life) on a scale of 0 to 10 [22] (see [Appendix E](#_Annex_E_:)). |
| Nervous system dysfunction drivers | Radicular signs/symptoms, hyperalgesia/allodynia, central sensitization | The ***PainDetect Questionnaire*** [23] is a reliable screening tool to predict the likelihood of a neuropathic pain component. The total score indicates if the pain is less likely to be neuropathic (negative, 0 to 12), uncertain (13 to 18) or like to be neuropathic (positive, 19 to 38) (see [Appendix F](#_Annex_F_:)).  The ***Central sensitization Index*** (CSI) consist of a self-reported tool to assess symptoms of central sensitization (CS) [24, 25]. It contains two sections, part A and B. Part A contains 25 items with 5-point Likert scale with a range for the total score from 0 to 100 and is intended to give an overview of the symptoms that are common in CS. Part B identifies if the patient has been diagnosed with specific disorders associated with CS as well as anxiety and depression (See [Appendix G](#_Appendix_G:_link)). |
| Cognitive-emotional drivers | False-beliefs, fear of pain/movement, illness perception, self-efficacy, mood, | The ***StartBackTool*** (SBT) is a screen­ing questionnaire consisting of 9 items based on psychosocial factors used to categorize patients with LBP based on risk (low, medium, or high) for poor disability outcomes [26]. Overall scores (ranging from 0 to 9) are determined by summing all responses, and the SBT psychosocial subscale (items 5-9; ranging from 0 to 5) are determined by summing all items related to psychosocial factors of prolonged disability such as catastrophizing and pain-related fear and anxiety [26] (see [Appendix H](#_Appendix_H:_link)). |
| Contextual drivers | Job flexibility, occupational factors | The ***Örebro Musculoskeletal Pain Screening Questionnaire*** (OMPSQ) is a 25-items questionnaire used to determine the risk of long-term absenteeism from work due to LBP *based on occupational and social factors* [27]. It also has a shorter 10-items version with comparable predictive properties to those of the long version while being easier to use in clinical settings [28] (see [Appendix I](#_Appendix_I:_link)) |
| Comorbidity drivers | Co-occurring MSK/mental health conditions, | From patient’s history [29] (At T2 only) |

**Timeline**

The timeline of thedata collection process is shown in figure 1 (See figure 1)

**Figure 1. Timeline of the feasibility study**

**
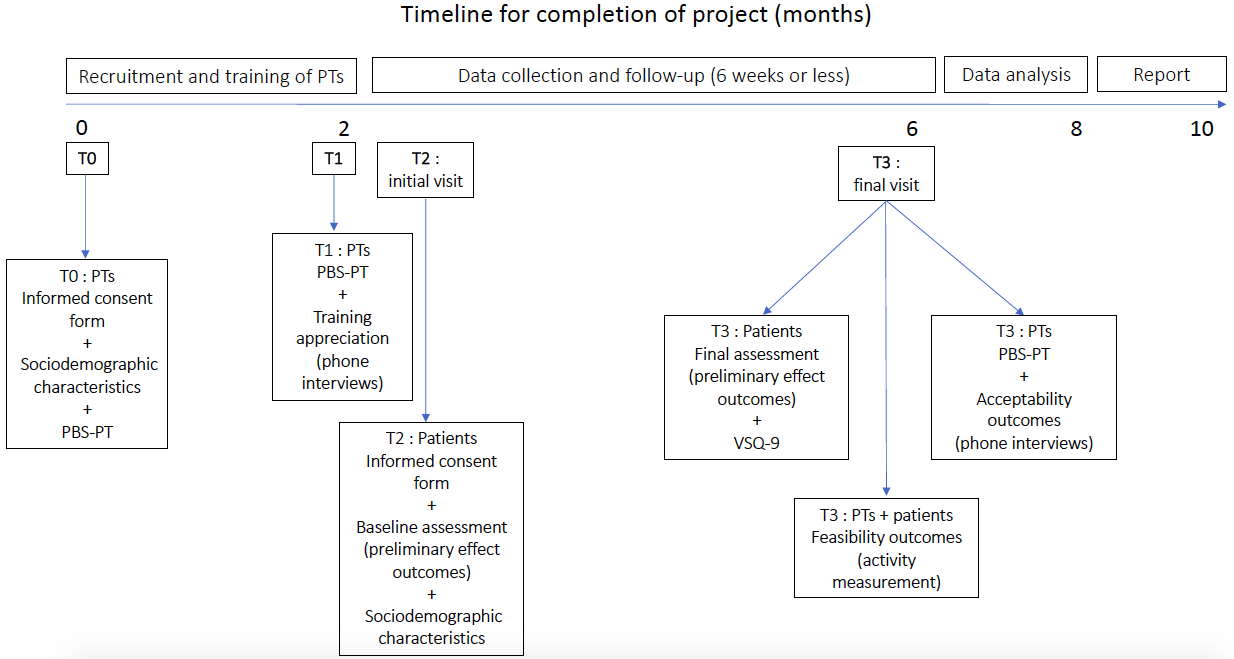
**

**Data analysis**

Baseline characteristics will be summarized for all participants (PTs and patients) using descriptive statistics. We are also going to use descriptive statistics to determine the percentage of patients that fall into the different subcategories of the model. Feasibility and acceptability outcomes will be assessed with quantitative descriptive statistics and qualitative reporting of data. Thematic analyses will be conducted on the verbatim transcripts of the semi-structured interviews in order to identify the predominant themes related to the PT’s acceptability of the training workshop and of the assessment procedures. As for the preliminary effect of the intervention on patient outcomes, we will calculate the change (delta score from T2 to T3) for each self-reported questionnaire.

**Ethical considerations**

Informed consent from all participants to participate in this study will be obtained. For the PTs, their consent will be obtained via a consent form handed to them at the beginning of the training workshop. Since the patients are already seeking and/or receiving healthcare services, only their consent to fill out questionnaire for the purpose of the study will be obtained by the PTs themselves via a second consent form with detailed information concerning the study process and their implication. In fact, the care received by the patients in this study is in the same continuum of standard care. They will be advised that all collected data would be kept confidential and anonymized by the use of alphanumeric coding only known to the researchers. Patients will receive a 20 $ Amazon electronic gift card to compensate for the time necessary to complete all the questionnaires at the time of the final visit (T3).

All electronic identifiable information will be held on a secure, password-protected database, accessible only to the members of the research team. Paper forms with identifiable information will be held in secure, locked filing cabinets within a restricted area. All paper and electronic data will be retained for at least 5 years after completion of the trial. There is no additional risk or inconvenient for the PT or the patient related to their participation in the study.

**References**

1. Balague F, Mannion AF, Pellise F, Cedraschi C, Balagué F, Mannion AF, et al. Non-specific low back pain. Lancet. 2012;379:482–91. doi:10.1016/S0140-6736(11)60610-7.

2. Donelson R, McIntosh G, Hall H. Is it time to rethink the typical course of low back pain? PM {&} R J Inj Funct Rehabil. 2012;4:394--401; quiz 400. doi:10.1016/j.pmrj.2011.10.015.

3. Driscoll T, Jacklyn G, Orchard J, Passmore E, Vos T, Freedman G, et al. The global burden of occupationally related low back pain: Estimates from the Global Burden of Disease 2010 study. Ann Rheum Dis. 2014;73:975–81.

4. Vos T, Allen C, Arora M, Barber RM, Brown A, Carter A, et al. Global, regional, and national incidence, prevalence, and years lived with disability for 310 diseases and injuries, 1990–2015: a systematic analysis for the Global Burden of Disease Study 2015. Lancet. 2016;388:1545–602.

5. Chou R, Shekelle P. Will this patient develop persistent disabling low back pain? JAMA. 2010;303:1295–302.

6. Taylor JB, Goode AP, George SZ, Cook CE. Incidence and risk factors for first-time incident low back pain: A systematic review and meta-analysis. Spine J. 2014;14:2299–319. doi:10.1016/j.spinee.2014.01.026.

7. Organization WH. International classification of functioning, disability and health. Geneva, Switzerland; 2001.

8. Escorpizo R. Defining the principles of musculoskeletal disability and rehabilitation. Best Pract Res Clin Rheumatol. 2014;28:367–75. doi:10.1016/j.berh.2014.09.001.

9. Karayannis N V, Jull GA, Hodges PW. Physiotherapy movement based classification approaches to low back pain: comparison of subgroups through review and developer/expert survey. BMC Musculoskelet Disord. 2012;13:24.

10. Rabey M, Smith A, Beales D, Slater H, O’Sullivan P. Multidimensional Prognostic Modelling in People with Chronic Axial Low Back Pain. Clin J Pain. 2017;33:877–91.

11. Rabey M, Beales D, Slater H, O’Sullivan P. Multidimensional pain profiles in four cases of chronic non-specific axial low back pain: An examination of the limitations of contemporary classification systems. Man Ther. 2015;20:138–47.

12. Bhise V, Rajan SS, Sittig DF, Morgan RO, Chaudhary P, Singh H. Defining and Measuring Diagnostic Uncertainty in Medicine : A Systematic Review. 2017;:22–5.

13. Tousignant-Laflamme Y, Martel MO, Joshi A, Cook C. Rehabilitation management of low back pain – it’s time to pull it all together! J Pain Res. 2017;Volume 10:2373–85. doi:10.2147/JPR.S146485.

14. Tousignant-Laflamme Y, Walton D, Wideman T, Lam O-T, Martel M, Cook C, et al. Operationalization of the new Pain and Disability Drivers Management Model: A Consensus study. 2018.

15. Eldridge SM, Chan CL, Campbell MJ, Bond CM, Hopewell S, Thabane L, et al. CONSORT 2010 statement: Extension to randomised pilot and feasibility trials. Pilot Feasibility Stud. 2016;2:1–32. doi:10.1186/s40814-016-0105-8.

16. Hro A, Mann H, Dickersin K, Berlin J, Dore C, Parulekar W, et al. Research and Reporting Methods Annals of Internal Medicine SPIRIT 2013 Statement : Defining Standard Protocol Items for Clinical Trials. Ann Intern Med. 2013;158:200–7.

17. Arain M, Campbell MJ, Cooper CL, Lancaster GA. What is a pilot or feasibility study? A review of current practice and editorial policy. BMC Med Res Methodol. 2010;10:1–7. doi:10.1186/1471-2288-10-67.

18. Sekhon M, Cartwright M, Francis JJ. Acceptability of healthcare interventions: An overview of reviews and development of a theoretical framework. BMC Health Serv Res. 2017;17:1–13. doi:10.1186/s12913-017-2031-8.

19. Abbott JH. The Distinction Between Randomized Clinical Trials (RCTs) and Preliminary Feasibility and Pilot Studies: What They Are and Are Not. J Orthop Sports Phys Ther. 2014;44:555–8. doi:10.2519/jospt.2014.0110.

20. Mutsaers JHAM, Peters R, Pool-Goudzwaard AL, Koes BW, Verhagen AP. Psychometric properties of the Pain Attitudes and Beliefs Scale for Physiotherapists: A systematic review. Man Ther. 2012;17:213–8. doi:10.1016/j.math.2011.12.010.

21. Visit-Specific Satisfaction Instrument (VSQ-9) | RAND. https://www.rand.org/health/surveys_tools/vsq9.html. Accessed 22 May 2018.

22. Tan G, Jensen MP, Thornby JI, Shanti BF. Validation of the Brief Pain Inventory for chronic nonmalignant pain. J Pain. 2004;5:133–7.

23. Freynhagen R, Baron R, Gockel U, Tölle TR. pain *DETECT* : a new screening questionnaire to identify neuropathic components in patients with back pain. Curr Med Res Opin. 2006;22:1911–20. doi:10.1185/030079906X132488.

24. Mayer T, Neblett R, Cohen H. The development and psychometric validation of the central sensitization inventory. Pain …. 2012;12:276–85. doi:10.1111/j.1533-2500.2011.00493.x.The.

25. Scerbo T, Colasurdo J, Dunn S, Unger J, Nijs J, Cook C. Measurement Properties of the Central Sensitization Inventory: A Systematic Review. Pain Pract. 2018;18:544–54.

26. Beneciuk JM, Bishop MD, Fritz JM, Robinson ME, Asal NR, Nisenzon AN, et al. The STarT back screening tool and individual psychological measures: evaluation of prognostic capabilities for low back pain clinical outcomes in outpatient physical therapy settings. Phys Ther. 2013;93:321–33.

27. L’heureux A, Berquin A. Comparison between the STarT Back Screening Tool and Örebro Musculoskeletal Pain Screening Questionnaire : Which tool for what purpose ? A semi-systematic review. Ann Phys Rehabil Med. 2019. doi:10.1016/j.rehab.2018.09.007.

28. Linton SJ, Nicholas M, Macdonald S. Development of a Short Form of the Örebro Musculoskeletal Pain Screening Questionnaire. 2011;36:1891–5.

29. Oostendorp RAB, Elvers H, Mikołajewska E, Laekeman M, van Trijffel E, Samwel H, et al. Manual physical therapists’ use of biopsychosocial history taking in the management of patients with back or neck pain in clinical practice. ScientificWorldJournal. 2015;2015:170463.

# Appendix A: invitation letter

Bonjour Madame/Monsieur,

C’est avec enthousiasme que nous communiquons avec vous, à titre de physiothérapeute travaillant auprès d’une clientèle présentant des troubles musculo-squelettiques, tels que la lombalgie mécanique non-spécifique. Nous souhaitons solliciter votre participation à une étude qui vise à déterminer la faisabilité d’implantation ainsi que les effets préliminaires d’un modèle de gestion des vecteurs de douleur et d’incapacité secondaire à la lombalgie non-spécifique nouvellement développé par l’équipe de recherche en milieu clinique. Vous recevrez cette invitation parce que votre milieu de travail a démontré un intérêt à collaborer sur ce projet de recherche.

Cette étude est menée par Yannick Tousignant-Laflamme, chercheur principal au centre de recherche du CHUS et directeur du programme de physiothérapie de l’École de la réadaptation de l’Université de Sherbrooke. Les co-chercheurs sont Marc O. Martel, Ph.D. (Université McGill), Simon Décary, Ph.D. (Université Laval), Dre Sylvie Lafrenaye, Ph.D.(Université de Sherbrooke), Lisa Carlesso, Ph.D. (Université de Montréal) ainsi que Christian Longtin, M.Pht (Université de Sherbrooke).

En bref, il s’agit de participer à une formation d’une durée d’une journée visant à vous familiariser avec le modèle de gestion des vecteurs de douleur et d’incapacité et acquérir des connaissances de bases quant à son application. Les objectifs de la formation sont :

- Définir les différents domaines du modèle et identifier les éléments spécifiques à chaque domaine
- Pour chaque domaine du modèle, choisir les bons outils afin d’établir le profil du client et documenter la présence d’éléments contribuant au portrait clinique
- À partir d’histoire de cas, adopter une approche structurée pour gérer et choisir les interventions appropriées afin d’adresser les domaines problématiques

Par la suite, il sera question de recruter environ 5 patients aux prises avec des douleurs lombaires et d’effectuer un suivi à l’aide du modèle présenté sur une durée maximale de 6 semaines lors duquel il vous sera demandé de rapporter une série de variables pertinentes à l’étude.

La rencontre aura lieu le .

Si cette participation vous intéresse, je vous invite à prendre connaissance du formulaire de consentement ci-joint. Celui-ci décrit l’étude, les critères d’admissibilité et en quoi consiste votre participation, si vous accepté de participer.

N’hésitez pas à me contacter ou contacter monsieur Longtin (par courriel :[christian.longtin@usherbrooke.ca](mailto:christian.longtin@usherbrooke.ca) ou par téléphone 819-346-1110, ext. 12844), si vous êtes intéressé à participer ou si vous avez des questions.

Merci pour votre précieuse collaboration.

# Appendix B : link to full content of training

[**https://www.dropbox.com/s/sz9jd1n7ejtg3v3/Annexe%20B_Formation_%C3%80%20LA%20RESCOUSSE%20DES%20PERSONNES%20SOUFFRANT%20DE%20LOMBALGIES%20COMPLEXES.pptx?dl=0**](https://www.dropbox.com/s/sz9jd1n7ejtg3v3/Annexe%20B_Formation_%C3%80%20LA%20RESCOUSSE%20DES%20PERSONNES%20SOUFFRANT%20DE%20LOMBALGIES%20COMPLEXES.pptx?dl=0)

# Appendix C – Questions for clinicians (training appreciation)

Appréciation de la formation de la part des cliniciens (guide d’entrevue téléphonique dans la semaine suivant la formation, T1) :

1. Est-ce que le fonctionnement du modèle, de façon générale, fait du sens à vos yeux ? Veuillez me détailler votre réponse.
2. Que pensez-vous de la quantité d’information contenue dans la formation ?
3. Que pensez-vous de la durée de la formation ?
4. Que pensez-vous du déroulement de la formation ?
5. Selon vous, quels sont les points forts/bons coups (ce qui vous a le plus aider) de la formation ?
6. Selon vous, quels seraient les aspects précis de la formation à améliorer ?
7. De manière générale, avez-vous des suggestions afin d’améliorer le déroulement général de la formation ?

Appréciation des ressources disponibles pour supporter l’utilisation du modèle dans la pratique des cliniciens (entrevue téléphonique, T3):

1. Est-ce que les ressources mises à votre disposition pour faciliter l’application du modèle dans votre pratique étaient suffisantes ?
2. Comment avez-vous trouvé l’utilisation des différentes ressources disponibles en termes de facilité d’utilisation, de pertinence et de disponibilité ?
3. Pensez-vous continuer à utiliser ces ressources et le modèle malgré la fin du projet de recherche (intégration à votre pratique?)

# Appendix D : link to Visit-Specific Satisfaction instrument

[**https://www.dropbox.com/s/ul7jyhpv4c6k6g0/Annexe%20D_satisfaction_patients_vsq9_adapted.pdf?dl=0**](https://www.dropbox.com/s/ul7jyhpv4c6k6g0/Annexe%20D_satisfaction_patients_vsq9_adapted.pdf?dl=0)

# Appendix E : link to Brief Pain Inventory

[**https://www.dropbox.com/s/nccv5egw3f6qkpm/Annexe%20E_Bref_Inventaire_Douleur_FR.pdf?dl=0**](https://www.dropbox.com/s/nccv5egw3f6qkpm/Annexe%20E_Bref_Inventaire_Douleur_FR.pdf?dl=0)

# Appendix F : link to Pain Detect Questionnaire

[**https://www.dropbox.com/s/spi48fexcwz0vd2/Annexe%20F_PainDetect_questionnaire_FR.pdf?dl=0**](https://www.dropbox.com/s/spi48fexcwz0vd2/Annexe%20F_PainDetect_questionnaire_FR.pdf?dl=0)

# Appendix G: link to Central sensitization inventory

[**https://www.dropbox.com/s/bxkg1uegkqlb2dc/Annexe G_CSI_fr.pdf?dl=0**](https://www.dropbox.com/s/bxkg1uegkqlb2dc/Annexe%20G_CSI_fr.pdf?dl=0)

# Appendix H: link to StartBack Questionnaire

[**https://www.dropbox.com/s/8906wj6wkugamzg/Annexe%20G_StartBack_FR.pdf?dl=0**](https://www.dropbox.com/s/8906wj6wkugamzg/Annexe%20G_StartBack_FR.pdf?dl=0)

# Appendix I: link to Orebro questionnaire

[**https://www.dropbox.com/s/nqqbqhql5wgx8dn/Annexe%20H_Orebro_version%20longue_FR.pdf?dl=0**](https://www.dropbox.com/s/nqqbqhql5wgx8dn/Annexe%20H_Orebro_version%20longue_FR.pdf?dl=0)
